# Central Giant Cell Granuloma of Posterior Maxilla: First Expression of Primary Hyperparathyroidism

**DOI:** 10.1155/2015/170412

**Published:** 2015-01-26

**Authors:** Deepanshu Gulati, Vishal Bansal, Prajesh Dubey, Sanjay Pandey, Abhinav Agrawal

**Affiliations:** ^1^Department of Oral and Maxillofacial Surgery, Subharti Dental College, Meerut 250005, India; ^2^Department of General Surgery, Subharti Medical College, Meerut 250005, India; ^3^Department of General Medicine, Subharti Medical College, Meerut 250005, India

## Abstract

A case of 19-year-old male patient reported with the chief complaint of slowly growing diffuse painless swelling over the right part of the face from last 6 months. Intraoral examination revealed a swelling on right side of palate in relation to molar region with buccal cortical plate expansion. Radiographic examination (orthopantograph and 3DCT) showed large multilocular radiolucency in right maxilla with generalized loss of lamina dura. Incisional biopsy was done and specimen was sent for histopathological examination which showed multinucleated giant cells containing 15–30 nuclei. Based on clinical, radiological, and histopathological findings provisional diagnosis of central giant cell granuloma was made. Blood tests after histopathology demonstrated elevated serum calcium level and alkaline phosphatase level. Immunoassay of parathyroid hormone (PTH) level was found to be highly elevated. Radiographic examination of long bones like humerus and femur, mandible, and skull was also done which showed osteoclastic lesions. Considering the clinical, radiographic, histopathological, and blood investigation findings, final diagnosis of brown tumour of maxilla was made. The patient underwent partial parathyroidectomy under general anaesthesia to control primary hyperparathyroidism. Surgical removal of the bony lesion was done by curettage. The patient has been followed up for 1 year with no postoperative complications and the lesion healed uneventfully.

## 1. Introduction

Central giant cell granuloma (CGCG) is a benign bone lesion which can be locally aggressive or may be asymptomatic in nature. In 1953, Jaffe described it as reparative granuloma of jaw bones [1]. But the term reparative is obsolete, as CGCG causes the destruction of involved bones. According to WHO, it is an intraosseous lesion consisting of cellular fibrous tissue that contains multiple foci of haemorrhage, aggregations of multinucleated giant cells, and occasional trabeculae of woven bone [2]. Females are more frequently affected than males with a sex ratio of 2 : 1 and more than 70% of lesions affect mandible while very few cases have been reported in respect to maxilla. Clinically these lesions cause facial swelling, asymmetry, and expansion of cortical plates and radiologically resorption of roots of teeth with cortical perforation is well appreciated [3]. Patients who present with a central giant cell lesion in the maxilla or mandible should be screened for hyperparathyroidism (HPT) to differentiate the lesion as a brown tumour. Although it is rare, CGCG of facial bones can be the first manifestation of HPT. There are 4 parathyroid glands located immediately behind the thyroid gland. They secrete parathormones (PTH) [4]. Normal levels of PTH ranges between 12 and 70 pg/mL [5] and excessive secretion of PTH causes a metabolic bone disorder known as HPT. Primary HPT results from autonomous hyperplasia or tumour, usually an adenoma. Secondary HPT develops in response to chronic low levels of calcium usually associated with chronic renal disease. Occasionally, parathyroid tumours develop after longstanding secondary HPT, known as tertiary HPT. A fourth type is ectopic HPT, which is thought to rise from increased PTH levels synthesized in patients with malignant disease [6]. Activity of parathyroid glands is regulated by free calcium levels in blood stream. Increased production of PTH causes an increase in serum calcium levels by decreasing renal tubular reabsorption of phosphorus. Alkaline phosphatase levels are increased and serum phosphatase values are decreased [7]. Thus, there is an imbalance between the osteoblastic and osteoclastic activity that causes resorption with fibrous replacement of the bone. Itonaga et al. indicate that a factor secreted by fibroblast/osteoblast (receptor activator of nuclear factor ligand (RANKL)) binds to stromal-monocyte derived cells to induce giant cell formation that might be reactive [8]. Studies conducted by Warnakulasuriya et al. [9, 10] have shown that, out of 300 patients with parathyroid tumours, 10 had facial bone involvement. Another study involving a group of 220 patients illustrates that only 4.5% of patients had lesion in jaws [11]. We present a case of CGCG of posterior maxilla in young adults that was found to be the first clinical manifestation of primary HPT.

## 2. Case Report

**Figure 1:**
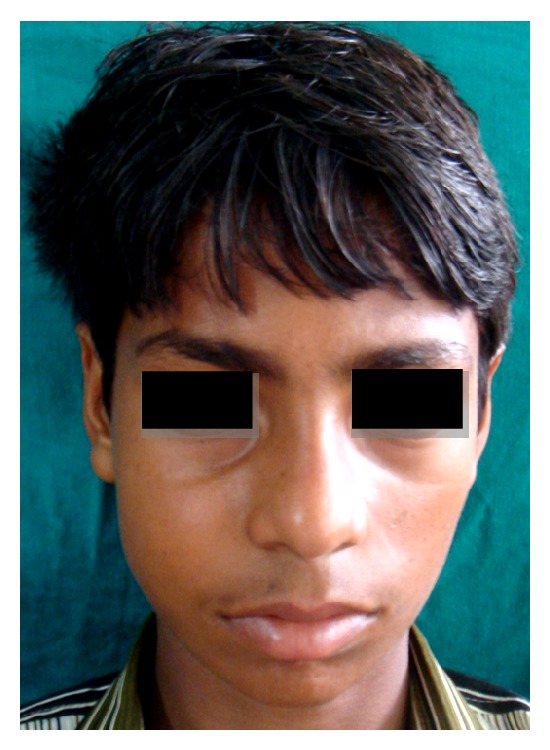
Swelling over the right cheek region.

**Figure 2:**
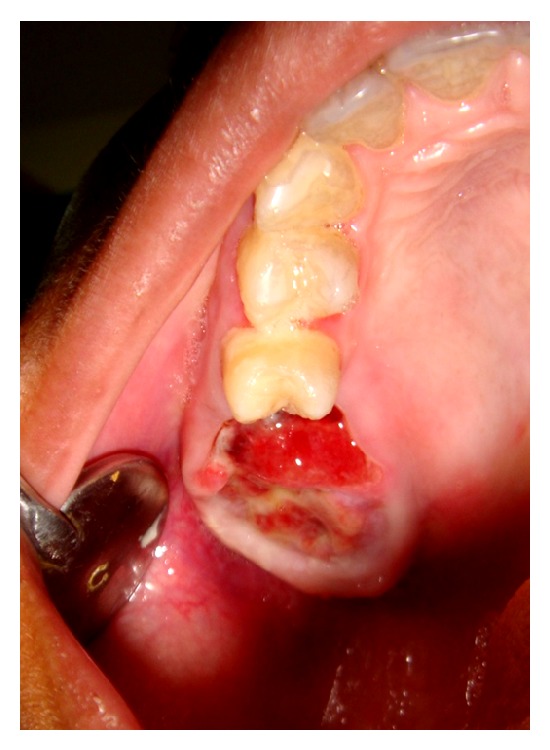
Intraoral photograph at first visit.

**Figure 3:**
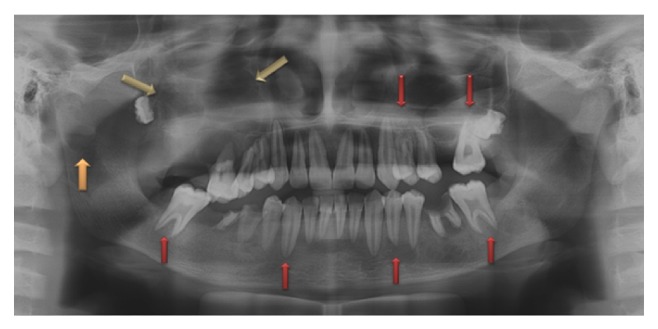
Preoperative radiograph represents multilocular radiolucency of maxilla, generalised loss of lamina dura, and unilocular radiolucency in condylar region.

**Figure 4:**
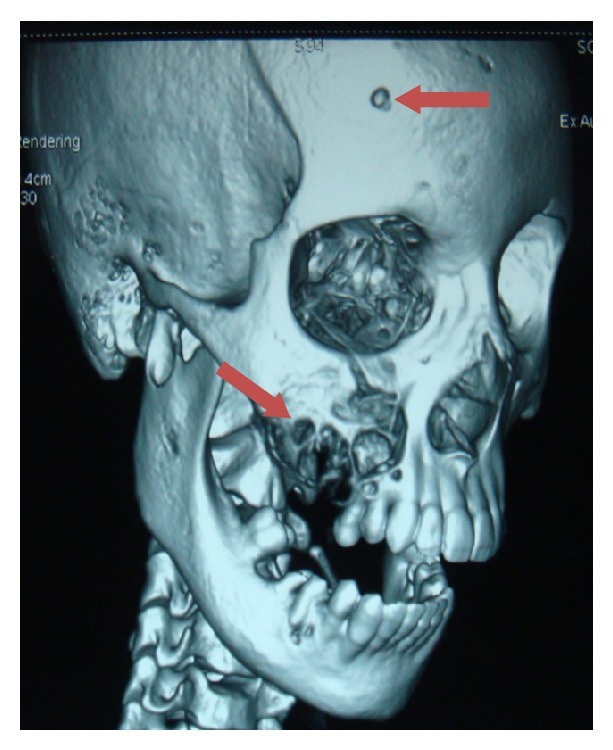
3DCT showing erosion of walls of maxillary sinus with destruction of alveolar process and osteoporotic changes in frontal bone.

**Figure 5:**
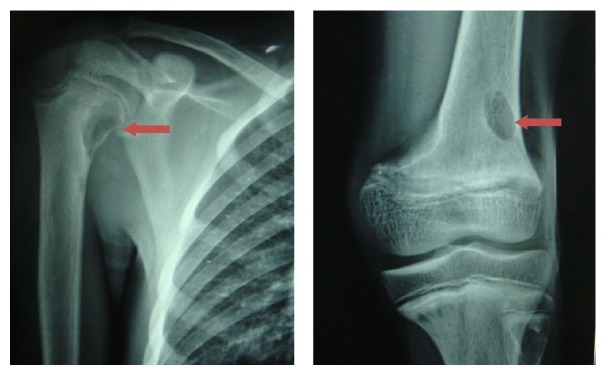
Osteoporotic changes in right humerus and left femur.

A 19-year-old young adult visited the outpatient clinic of the Department of Oral Surgery at Subharti Dental College with swelling over the right part of face from the last 6 months (Figure 1). The patient had undergone tooth extraction 6 months back. After 3-4 days, there was presence of swelling. Initially swelling was small in size, approximately 1 cm in diameter. There was no tenderness. Then there was gradual increase in size of swelling. The patient went to a nearby doctor where medications were prescribed. But there was no relief. Extraoral examination revealed facial asymmetry due to diffuse asymptomatic swelling starting from the lateral corner of the mouth and extending up to angle region of the same side. There was no change in colour of skin with any increase in temperature of overlying skin. On palpation, lymph nodes were not palpable. Intraorally, inflamed lesion with distinct border presented distal to the second premolar extending up to maxillary tuberosity region, approximately 2 cm in size. There was expansion of buccal cortical plate with expansion of palatal bone (Figure 2). The adjacent mucosa was normal in colour. All teeth were vital and the nerve sensibility was not disturbed. Panoramic radiography showed a multilocular radiolucent lesion suggestive of osteolysis in the right maxilla region. The lesion was extended from the root of the right upper canine to the maxillary tuberosity region and caused resorption of the first maxillary molar root. Unilocular radiolucency was also observed below the condylar process and frontal bone. Another prominent feature which was observed was generalised loss of lamina dura (Figure 3). Based on clinical and radiographic findings, differential diagnosis of CGCG, ameloblastoma, and odontogenic myxoma was made. Later a computed tomography was advised which showed a large expansible lesion in right maxillary sinus causing erosion of its walls causing destruction of alveolar process of upper maxilla compressing and bulging into the right nasal cavity (Figure 4). To further confirm the diagnosis, long bone radiographs were advised which showed osteoporotic lesion in right humerus and left femur bone (Figure 5). Generalized loss of lamina dura, maxillary alveolar bone demineralization, and multiple osteoporotic lesions in long bone are highly suggestive of brown tumour. Ideally parathyroid assay needs to be assessed before biopsy. But, due to financial constraint and availability of limited resources in our centre, biochemical investigations like PTH levels were not able to be performed before the biopsy.

**Figure 6:**
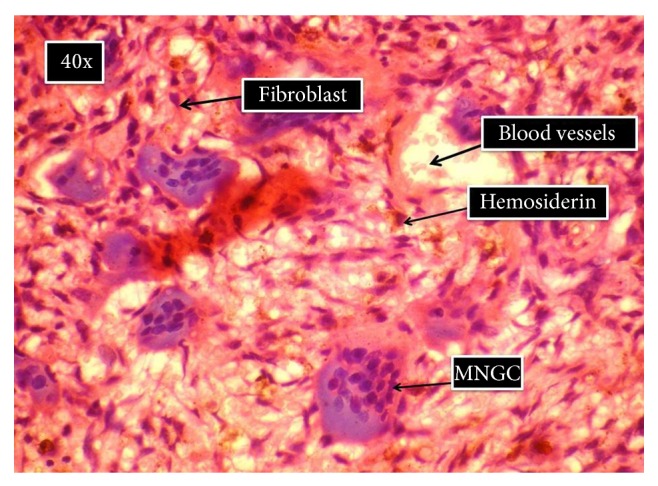
Histologic section showing connective tissue stroma, proliferating fibroblast, multinucleated giant cells, and interspersed hemosiderin pigment.

To confirm initial diagnosis, incisional biopsy was performed from maxillary lesion. Histopathology revealed numerous multinucleated giant cells within loose fibrillar connective tissue (Figure 6). Multinucleate giant cells are variable in size and shape containing nuclei ranging from few to 25 in numbers. Numerous foci of extravasated RBC with hemosiderin pigments were seen. Blood tests after histopathology demonstrated elevated serum calcium level 13.8 mg/dL (normal: 8.5–10.5 mL/dL) and alkaline phosphatase level 494 U/L (normal: 36–141 U/L). The immunoassay of PTH level by chemiluminescence method was elevated to 789.5 pg/mL (normal: 12–65 pg/mL).

**Figure 7:**
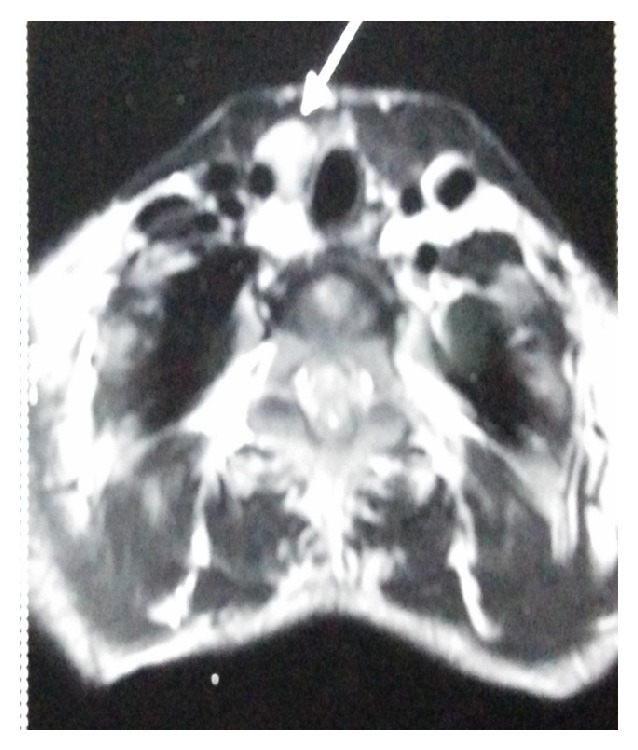
MRI showing lobulated lesion at the inferolateral aspect of right lobe of thyroid gland.

**Figure 8:**
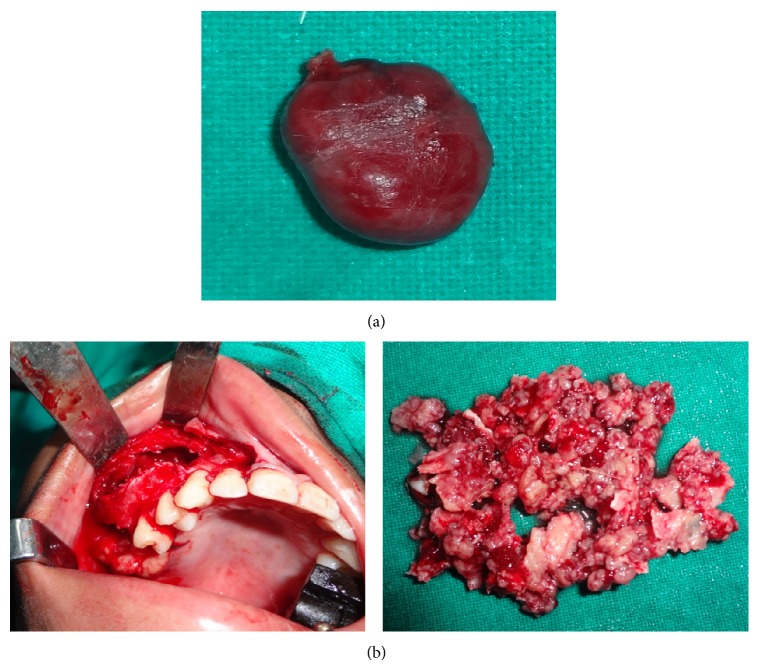
(a) Excised parathyroid adenoma. (b) Intraoperative photograph with excised curettage material.

**Figure 9:**
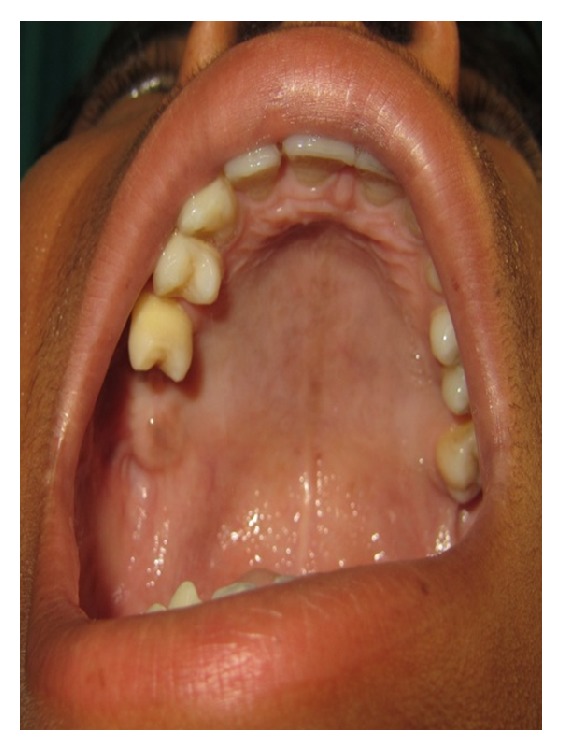
Regression of maxillary lesion.

**Figure 10:**
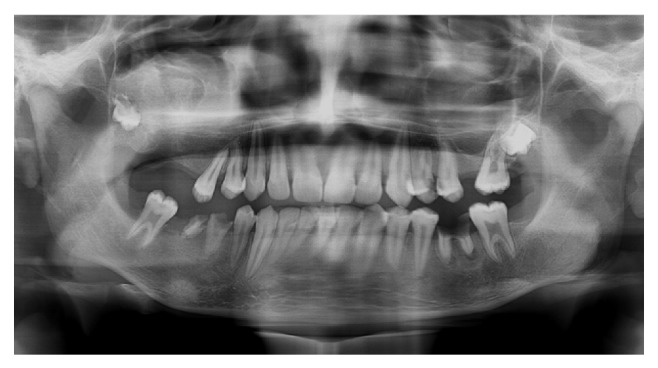
Postoperative radiograph showing signs of calcification in maxilla and condyle.

**Figure 11:**
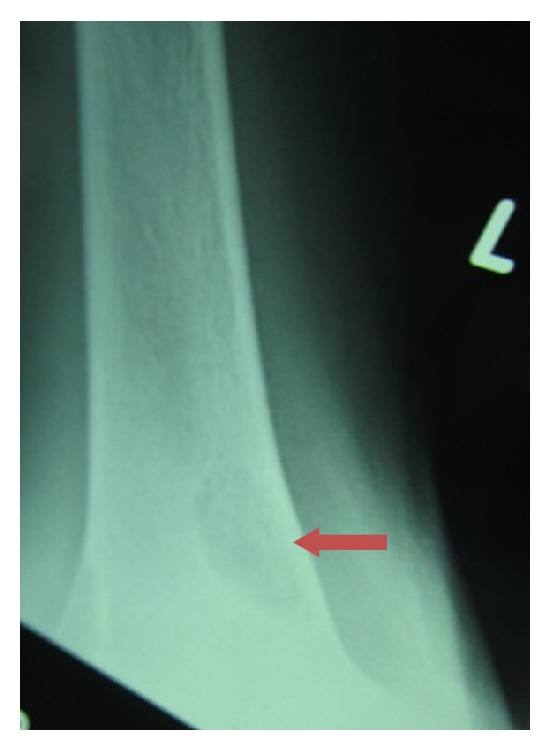
Sign of calcification in the left femur.

To assess the parathyroid gland, MRI of neck was advised which revealed small lobulated hyperintense lesion size 13.5 × 12.5 mm at the inferolateral aspect of the right lobe of parathyroid gland (Figure 7). Considering the clinical, radiographic, histopathological, and haematological findings, we arrived to the final diagnosis of brown tumour of maxilla secondary to primary HPT. Surgical treatment of the pathology was planned under general anaesthesia. Three days prior to surgery, to optimize calcium levels, zoledronic acid (bisphosphonate) in 100 mL normal saline was administered intravenously over 15–30 minutes. 3 litres of normal saline was also administered daily to maintain hydration. Along with right inferior lobe parathyroidectomy to control primary HPT, surgical curettage of maxillary lesion was performed with primary closure under one-time general anaesthesia (Figures 8(a) and 8(b)). On histopathological examination, excised gland was suggestive of adenoma and curettage material from right maxilla was consistent with CGCG. No postoperative complications occurred and the lesion healed uneventfully (Figure 9). Oral calcium supplementations in addition to Vitamin D3 were also prescribed for possible postoperative hypocalcaemia. The patient has been followed up from the last 1 year and there has been regression in maxillary swelling. Postoperative radiographs were advised after every 6 months which show signs of calcification and remodelling (Figures 10 and 11).

## 3. Discussion

The central giant cell lesion of the jaw is a rare benign tumour with an unknown aetiology accounting for up to 7% of tumours in the mandible (lower jaw) and the maxilla (upper jaw) [3]. As mentioned earlier, it can be aggressive or nonaggressive. According to Chuong et al. [12], aggressive giant cell lesions were defined as lesions exhibiting a size equal to or greater than 5 cm as well as showing rapid growth, tooth displacement, root resorption, cortical bone thinning, or perforation or recurrence after curettage. High recurrence rate has been reported with aggressive lesions as compared to nonaggressive lesions. But histologically there is no difference.

Management of CGCG includes surgical curettage with or without medical management. Medical therapies include intralesional corticosteroids, calcitonin injections, and interferon-alpha therapy. They are alternatives as well as adjuncts to surgical treatment.

In 1988, Jacoway et al. [13] first reported the treatment of CGCG with intralesional corticosteroid injections. 4 patients were included in the study and a weekly injection of triamcinolone acetonide into the lesion over a period of 6 weeks. There was complete resolution in 3 patients while additional surgical management was vital in 1 patient. Other authors also gave encouraging results with corticosteroid injections. In 1994, Kremer et al. administered triamcinolone 10 mg/mL and lignocaine 0.5% in 1 : 1 ratio once in a week for 5 weeks. Patients were followed up for 3 years with no remission. Similar concentration was used by Rajeevan et al. and patients were followed up for 10 months with no recurrence [14]. It is hypothesized that the extracellular production of bone resorption mediating lysosomal proteases by the giant cells is inhibited and steroids induce apoptosis of osteo-like cells [15]. It appears to work more successfully in unilocular lesions than multilocular lesions and is contraindicated in pregnant and immunosuppressive patients.

In 1993 Harris proposed the use of calcitonin. It increases influx of calcium into the bones and thus functions antagonistically to parathyroid hormone [15]. Synthesis and secretion of calcitonin occur in the parafollicular cells or C cells, lying in the interstitial fluid between the follicles of the thyroid gland. Calcitonin decreases blood calcium ion concentration by decreasing the absorptive activities of the osteoclasts and thus shifting the balance in favour of calcium deposition [4]. It also diminishes the formation of new osteoclasts. It has also been confirmed that giant cells express calcitonin receptors. Two types of calcitonin are used for the treatment of CGCG, salmon calcitonin and human calcitonin. At present salmon calcitonin is commercially available. Subcutaneous injection of 100 units of calcitonin daily up to 15 months is recommended while nasal spray with higher dose of 200 IU has better patient compliance [13]. Like intralesional steroids, successful studies had been reported in respect to calcitonin injections. In 1999, de Lange et al., reported complete remission of lesion when patients were followed up for 12–15 months and 19–21 months, respectively [16]. Radiographically, resolution does not normally commence until six to nine months of treatment and treatment is continued for up to 24 months to see the maximum resolution.

Another possible modality which is used for management of CGCG is interferon-alpha. Interferon is an antiviral and antiangiogenic agent that is used in conditions like infantile hemangiomas. It is produced by recombinant DNA technology or is purified from cultured human cells. Based on the assumption that this lesion may be vascular in origin, subcutaneous interferon-alpha has been used in the treatment of this lesion [17–19]. It is given for its antiangiogenic effects, though there is little evidence that the lesion is vascular in origin. The treatment has shown some success, but its use is limited by its side effects, which include headaches and a flu-like illness, with a number of patients having to be admitted to hospital for management.

The giant cells in CGCG are osteoclasts. Their formation involves the interaction between stromal cells, which express RANKL which is also a potent stimulator of osteoclast bone resorbing activity. These processes are inhibited by osteoprotegerin (OPG), which is a decoy receptor for RANKL, and also by a monoclonal antibody to RANKL (AMG 162). This indicates that osteolytic expansion in CGCG can theoretically be controlled by therapeutic agents that inhibit the RANKL/RANK interaction, such as OPG and AMG162.

At present, OPG and AMG162 are being evaluated for therapeutic use, mainly in studies in postmenopausal women. These reports indicate that both OPG and AMG 162 are rapidly effective and profoundly reduce bone turnover for a sustained period.

In another study, the effect of imatinib on osteoclasts was examined and it was found that there was a dose dependent decrease in RANK. This finding strongly suggests that imatinib may be an effective antiosteolytic agent and could therefore be useful in the treatment of skeletal diseases involving excessive osteoclast activity, such as CGCG [14].

Surgical curettage is the treatment of choice for CGCG. For aggressive lesions, en bloc resection is the treatment of choice. Even though radical resection is effective modality for aggressive lesions, it leads to functional disturbances [20].

CGCG in the upper jaw in the event of endocrine disorders is termed “brown tumour.”

In our case CGCG was the first manifestation with primary HPT which was confirmed by biochemical and radiological findings. Therefore, apart from CGCG, primary HPT was needed to be addressed.

There are different schools of thoughts according to literature for management of CGCG associated with HPT. Regression and healing of the lesions are expected after the correction of HPT. The proper timing of parathyroidectomy and its favourable effect on regression of the brown tumour made it possible to avoid a potentially disfiguring surgical removal of the brown tumour as reported by Di Daniele et al. [21].

There are many reasons why parathyroidectomy should be recommended for all patients with asymptomatic mild primary hyperparathyroidism. Untreated patients have a small risk of developing renal failure in the long term and an increased risk of bone loss particularly in older women. Palmer et al. conducted a cohort study and during 14-year follow-up found higher mortality rate with untreated hypercalcaemia than in age and sex matched controls. In primary HPT, there is an increased incidence of cardiovascular disease and it is predominant cause of death. Thus increased mortality can be reduced eventually by parathyroidectomy [22].

But several cases have been reported of brown tumours that grow and do not resolve after parathyroidectomy or normalization of PTH levels that further require resection of brown tumour [23].

Kennett and Pollick reported the need of surgical intervention for brown tumour even after parathyroidectomy because of slow regression which may take longer duration, even more than 5 years [24].

Cases have been reported where brown tumours continued to grow although patients had been normocalcemic 2-3 months and 2 years after parathyroidectomy. It is therefore recommended that, in such cases where resolution is slow or growth continues, local curettage or enucleation should be undertaken [25].

Both aspects of management were discussed with the parents of our patient; they agreed for simultaneous curettage along with parathyroidectomy under general anaesthesia due to financial constraint. Finally it was decided to perform surgical curettage via intraoral (I/O) approach along with parathyroidectomy, assuming that I/O approach will not cause much of functional and cosmetic deformity compared to aggressive resection via extraoral approach. Although the patient had existing missing upper right posterior teeth, later prosthetic rehabilitation for functional correction will be planned once the lesion healed satisfactorily. The patient was kept under regular follow-up and it was noticed that complete regression of swelling occurred after 1 year without any cosmetic deformity.

Parathyroid adenomas are usually small and almost never palpable in the neck. When clinical manifestations are present, the signs and symptoms can be divided into two types: urologic changes secondary to hypercalcemia and skeletal changes. Urologic changes include polyuria, polydipsia, and the development of kidney stones. In severe cases it may cause anorexia, vomiting, constipation, fatigue, weight loss, muscle weakness, psychiatric symptoms, and pancreatitis [26]. Except for muscle weakness and fatigue, other symptoms were absent in our case. Skeletal changes may represent the first manifestations of the disease, with a loss of cortical bone and an increase in trabecular bone. Bone pain and arthralgia are the most common symptoms. Radiologically, parathyroid adenomas may or may not exhibit well-defined margins. It may exhibit hyperparathyroidism-induced bone changes, such as medullary bone demineralization of the mandible [27]. Expansion of cortical plates and osteoporotic changes in maxilla, condylar process, and skull with loss of lamina dura were the first manifestations in this case.

## 4. Conclusion

Early diagnosis of HPT can be done with assessment of all the radiographic, biochemical, and histopathological parameters. Giant cell lesions like CGCG must be suspected and investigated to rule out hyperparathyroidism. Although the lesion resolves spontaneously after parathyroidectomy, surgical curettage should be well thought out for large lesions irrespective of site of lesion.

## Abbreviations

CGCG: Central giant cell granuloma
HPT: Hyperparathyroidism
PTH: Parathyroid hormone.
